# The Performative is Political: Using Counter‐Storytelling through Theater to Create Spaces for Implicated Witnessing

**DOI:** 10.1002/ajcp.12493

**Published:** 2020-12-22

**Authors:** Christina Maxwell, Christopher Sonn

**Affiliations:** ^1^ Institute for Health and Sport Victoria University Melbourne Victoria Australia

**Keywords:** Community arts, Counter‐storytelling, Implicated witnessing, Spaces of encounter, Whiteness

## Abstract

Performative counter‐storytelling can be a powerful experience for both the artists who create these stories and the audiences who witness them. This study examined audience responses to a counter‐narrative (entitled “AMKA”) performed by Africans in Australia which intended to present more complex, holistic, and strengths‐based representations of their communities than those currently circulated by dominant discourses. Guided by a critical whiteness lens, the study explored how 34 self‐identifying white audience members interpreted the performance and how they questioned whiteness by assuming the role of implicated witnesses. Following thematic analysis of mixed closed‐ and open‐ended post‐performance survey responses, audience members made connections between the content of AMKA and the contemporary political and cultural contexts in which it was performed and began to examine their positions of privilege and power. This study has provided evidence for the potential of political theater in creating spaces of encounter whereby responsible listening positions can be nurtured in the journey toward dismantling personal, and potentially structural, racially‐based injustices.

## Highlights

- Community arts can be a powerful method of engaging privileged groups in social justice.
- We investigate how white spectators interpret African Australians’ performative counter‐narratives.
- Surveys show how implicated witnesses are engaged in critical reflection of dominant narratives.
- Political theatre creates spaces to examine and question whiteness using embodied knowledges.

## Introduction

The creation of, and participation in, the arts is increasingly being recognized as an important medium for achieving social justice above and beyond its capacity for providing transient esthetic pleasure. Community arts practices have already been applied to a range of injustices to enact change from the individual to the community and with the potential to support enduring systemic transformation. Such settings include: mental health (Faigin & Stein, 2015; Mohatt et al., 2013), migration (Baker, Sonn, & Meyer, 2020; Beauregard et al., 2020) racial profiling (Sonn, Quayle, Belanji, & Baker, 2015), lesbian, gay, bisexual, transgender, and queer activism (LGTBQ; Wernick, Kulick, & Woodford, 2014), homelessness (de Oliveira, 2019), and intimate partner violence (Sajnani, 2010) to name a few. Although artistic expression can take many forms, the focus of the current research is on examining how theater can bring silenced and oppressed narratives into public awareness which, in turn, can facilitate critical reflexivity among the audiences who witness them. Specifically, this study collected audience data from a performance in Melbourne called “AMKA”, an artistic communication of a counter‐narrative created by Africans in Australia^1^ in response to the public narrative. These data were used to elucidate understandings about how spaces for encountering difference are navigated within the theater setting.

### Community Arts for Social Change

The arts have long been acknowledged as a contributor to social value with a study conducted in 2014 by the Australian Council for the Arts (2014) reporting that the arts: allow for expression and creativity, deliver positive outcomes for child development, and increase mental health and wellbeing. Within community psychology research and practice, artistic methods have been employed to promote: critical thinking skills (Fernández, 2018a; Lea, Malorni, & Jones, 2019), self‐determination and empowerment (Wernick et al., 2014), social activism (Faigin & Stein, 2015), resilience (Lea et al., 2019), social connectedness and belonging (Baker et al., 2020; Beauregard et al., 2020), culturally relevant evaluation practices (Straits, deMaría, & Tafoya, 2019), and collective memory and healing (Mohatt et al., 2013; Quayle, Sonn, & Kasat, 2016).

The community arts are also a powerful way to engage audiences in difficult and confronting conversations, with Gergen and Gergen (2011) commenting that the arts “can more effectively motivate interest and action, and they can enhance dialogues on important societal issues” (p. 295). To illustrate, adult attendees of a photovoice exhibition developed by LGBTQ youth reported increased awareness about LGTBQ issues and confronted their own biases and use of non‐inclusive language (Hall, Witkemper, Rodgers, Waters, & Smith, 2018). The power of socially engaged art is connected with performative inquiry that embraces diverse methods and modes of representation and action necessary for social and cultural impact (Gergen & Gergen, 2011). Critical community psychologists have also advocated for decolonial approaches to research and action that can bring the field closer to its goals of liberation and epistemic and social justice (Fine, 2018; Sonn & Baker, 2016; Straits et al., 2019; Teo, 2018).

### Performative Counter‐Storytelling

One frequently employed method of artistically communicating social justice issues is performative counter‐storytelling. Defined as the presentation of narratives that challenge the validity and completeness of those available in the public domain, counter‐storytelling intends to dismantle overlearned, reductionist, and oppressive representations of marginalized people while offering balanced and more complex accounts of experience (Solórzano & Yosso, 2002). These biased representations, or “dominant cultural narratives” (Rappaport, 1995, p. 803), are uncontested, entrenched, and perpetuated through institutions and everyday interactions to legitimize some bodies and vilify others. Performative counter‐storytelling is concerned with challenging and replacing these narratives with alternatives that are empowering, inclusive, and self‐authored by the communities that they represent, functioning as an “aesthetic of interruption” (Baker et al., 2020, p. 15) to the dominant narrative.

When combined with artistic approaches, counter‐storytelling can open dialogue and discussion, present and celebrate alternative ways of knowing, challenge one‐dimensional perspectives of relating to minority group members, and remember and archive the past while contemplating “counter‐possibilities” for the future (Martínez, 2017, p. 112). Quayle et al. (2016) have used counter‐storytelling through portraiture to disrupt public memory around silenced Aboriginal narratives by displaying paintings of Noongar Elders in an exhibition space in Western Australia. Further, Harter, Scott, Novak, Leeman, and Morris (2006) document how Passion Works in Ohio combines counter‐narratives with a variety of art mediums to oppose dominant cultural narratives of individuals with lived experience of disability and instead portray themselves as autonomous, agentic, and holistic. Although art creation in and of itself can provide positive effects for artists, socially engaged art necessitates a receptive audience. The audience, however, is not a homogenous entity; Hughes‐Hassell (2013) illustrates the differences in the impacts among spectators by describing performances as being like a mirror for those with shared experiences with the artist (e.g., ethnic, gender, or religious identity) and as being like a window for those without these experiences. Similarly, both Quayle et al. (2016) and Martínez (2017) report how the marginalized groups in their research wanted their art and their narratives to reach audiences with unexamined privilege to increase education and awareness about the former.

However, both Watt (2007) and Thompson (2003) note that defensive reactions are to be expected when one is confronted with their complicity in an oppressive system. When one centers their guilt, anger, and anxiety and prioritizes the alleviation of these emotions over and above deconstructing their relationship to whiteness and the structures that uphold it, they re‐enact performances of privilege. As such, in order to be effective allies, Watt and Thompson argue that these reactions should be acknowledged and explored constructively rather than met with denial or a paralysis from action. Community art approaches are one such tool that can be employed to stimulate these conversations as, by nature, they are well positioned to engage the emotive and non‐verbal which may not be accessible via cognitive expression.

### Theater as a Space of Encounter

Theater is one means of communicating counter‐narratives in an accessible and engaging manner. By bringing together artists and audiences who experience different levels of access to power, political theater can help cultivate both a physical and symbolic “space of encounter” (Mayblin, Valentine, & Andersson, 2016, p. 216) in which intercultural exchange is facilitated and social distance is reduced (Sajnani, 2012). These spaces can be distinguished from “contact zones” (Pratt, 1991, p. 34) which are often used in community art projects to bring together culturally diverse groups to co‐develop an art piece (e.g., Beauregard et al., 2020; Madyaningrum & Sonn, 2011; Mohatt et al., 2013). Specifically, contact zones are defined by ongoing interactions between individuals with varying lived experiences while spaces of encounter are singular and temporary events, much like one would expect of an artist‐audience interaction at a theater performance. The latter has been found as an effective way to foster growth‐oriented encounters, such that audiences can engage constructively with difference while critically reflecting on their own power, privilege, and positioning. For instance, Mulvey and Mandell (2007) demonstrate how community audiences of The Laramie Project in Lowell, Massachusetts came to question their behaviors toward intolerance and the structures that sanction injustice toward LGBTQ people.

So, what happens in this in‐between space, this space of contestation, unfamiliarity, and encounter? In art theory more broadly, Conner (2004) suggests that it is within this space that an artwork is completed; an artist creates an artwork with an intention of what they want to convey to their audience, and yet it is the unique interaction between the audience and the art that gives way to a multitude of possibilities for interpretation. In this way, art is inherently relational, depending on the coming together of artists, audiences, and context to imbue art with meaning. Teo (2016) argues that it is in this space of connection between people who occupy different social locations that dominant cultural groups can become aware of their privileges which are otherwise left unexamined and beyond awareness.

Fine, Weis, Centrie, and Roberts (2000) posit that the act of connecting across difference is educating and that learning is often inherent in intercultural exchanges. Although encounters of diversity and subsequent opportunities for meaningful dialogue may occur in formal institutions such as classrooms, it is argued that more complete knowledges must be sought elsewhere. At its most fundamental level, public pedagogy refers to the ways in which learning occurs outside of formal educational institutions (Sandlin, O'Malley, & Burdick, 2011). Through the use of public spaces as sites for accessible, unstructured, and uninhibited education, transformational learning and unlearning can occur. In this way, the learning in these alternative spaces is not limited to traditional means of information dissemination through written text; it is also non‐verbal and embodied (Ellsworth, 2004).

One does not just “find” themselves in these spaces, however; they often need to actively seek them out. As such, those who pursue and attend sites of art‐based intercultural dialogue are likely to already have a foundation of critical reflection skills. Although it is important for these individuals to remain engaged, there is a larger discussion to be had as to how artists reach those who most need to hear the counter‐narrative. That is, as meaningful engagement largely relies on one’s own initiative and level of interest, how can “receptive social environments” (Campbell & Cornish, 2012, p. 847) be nurtured among those who are resistant to entering these conversations?

Sajnani (2012) contemplates what might happen if these people were sought out and invited to join these spaces. She suggests that connecting culturally diverse artists and audiences can be achieved via active participation in the arts by the latter, such as is characteristic of Boal’s (1985) “Theatre of the Oppressed” techniques. Conversely, Lord (2012) argues that audience members do not need to have an active role in production in order to participate in, and be transformed by, the arts. Instead, a more appropriate descriptor for those spectators who are not explicitly involved in co‐production may be “witness.” Far from being an indifferent bystander, witnesses are attentive, reflective, and critically engaged listeners (Sajnani, 2012; Stein & Mankowski, 2004). They are “implicated” (Sajnani, 2012, p. 6) in social justice by an action‐oriented sense of responsibility and accountability to those whom they have oppressed. For instance, by relying solely on the sensory modality of sound, Baker et al. (2020) demonstrate the potential for artistic approaches to transform inactive observers into responsible and empathic listeners through their documentation and public presentation of South African migrant narratives.

For the purposes of the current study, to be an implicated witness is to experience an alteration of the relationship between the spectator and the performer as well as a shift in self‐knowledge for the former. Ideally, this transformation comes in the form of a “psychic revolt” (Bell, 2016, p. 288) that awakens one into responsiveness via critical reflection and questioning. As such, “awakening” (Watkins & Shulman, 2008, p. 233) involves increasing one’s awareness of this privilege, which may occur many times across a lifetime of education, reflection, and action. However, whether or not audience members occupy this role of a witness in the performance space can be dependent on their positioning in relation to the artists. That is, race, gender, and other dimensions of power may shape how people make sense of encounters through theater.

### Whiteness as a Lens for Inquiry

Although art has been used as a bridge for understanding between a range of culturally diverse groups (e.g., Harter et al., 2006; Wernick et al., 2014), performative counter‐storytelling has principally been used between racially/ethnically marginalized artists and dominant cultural audiences (e.g., Martínez, 2017; Quayle et al., 2016; Sonn, Quayle, Mackenzie, & Law, 2014). Those in dominant cultural positions are implicated as they are afforded privileges at the expense of all others who are instead positioned against and below the normative whiteness (Reyes Cruz & Sonn, 2011). The notion of whiteness is a metaphor for unearned privileges and the normativity of one’s culture in the context of racialized social relations. Such privileges include racially encoding space to afford whiteness comfort in learned expansiveness, that is, uncontested access to any space (Sullivan, 2006), assuming narrator roles that skew collective remembering and knowledge production (Fernández, 2018b), and indulging in a white ignorance that de‐contextualizes racial injustices (Coleman, Collins, & Bonam, 2020). These colonial structures of whiteness often operate insidiously and are reinforced historically, politically, and socially.

Coleman et al. (2020) have recently called for community psychology to better incorporate critical whiteness studies into the field. A critical whiteness lens implicates white audiences in examining racialized subjectivities as issues of race are typically considered a “non‐white” problem or, when acknowledged, responsibility for change is displaced onto those who are explicit and extreme in their racism (Green, Sonn, & Matsebula, 2007). Ultimately, Mezirow and Taylor (2009) argue that a commitment to antiracism as a white person is a lifelong journey that requires humility in acknowledging one’s ignorance as well as a deliberate effort to engage in risk, vulnerability, and discomfort. It also requires individuals to assume responsibility for their own unlearning of internalized white supremacy without the need for external validation or other incentives. It is argued in the current paper that political theater can act as a conduit for these conversations that “disrupt white innocence” (Fernández, 2018b, p. 294) by providing opportunities that facilitate “the ability to turn the lens inward, to self‐evaluate, critique, and discern colonial ways of thinking and being” (Fernández, 2018b, p. 297).

### Community Theater: AMKA

In Melbourne in particular, the dominance of white discourse and practices is most prevalent when considered alongside the subsequent oppression of the African Australian community. Specifically, moral panics have been fueled by the media to position those perceived as African as violent, delinquent, and dissimilar to the white majority (Windle, 2008). In addition, a 2016 Scanlon Foundation report (Markus, 2016) found that an average of 54% of African Australian migrants experience physical and verbal abuse, work discrimination, and exclusion from Australian society. There have been some attempts at facilitating intercultural exchange between white Australians and African Australians through the mediums of radio and theater (Budarick & Han, 2015; Sonn et al., 2015, respectively), yet more work remains to be done to examine the processes and outcomes of performance art for white audiences.

AMKA (previously “Afrobeat”) was an initiative of the cohealth Arts Generator (cAG) that used theater to depict the African diaspora in Australia. Translated from Swahili, AMKA means “get up” and communicates a sense of urgency. The central creative team involved in developing and performing AMKA was comprised of nine men and women of African background who created a multimodal art piece (consisting of music, spoken word, theater, dance, and projection) that was reflective of their individual stories and their collective narrative. These accounts sought to challenge the dominant narratives that frame their community in Australia while presenting alternative knowledges situated within historical, political, and intergenerational discourses.

The intention of the artists was to create an art piece that spoke to their heritage communities and to counter these prevailing narratives with ones that are complex, strengths‐based, and diverse. Even though the effects on audiences outside of those members of the African diaspora were considered incidental, the public nature of the event meant that a range of people were in attendance. As such, AMKA provided an opportunity to investigate the impacts of performative counter‐storytelling for those who are ostensibly white by examining what insights their reflections provided into the meaning‐making processes of the esthetic encounter.

### Overview of the Current Study

The present research intended to examine what it meant for white attendees to inhabit the temporary identity of “audience member” for the AMKA performance and how this identity impacted on their relationships with their whiteness. It also intended to alleviate the current gap in the literature about how community arts are experienced from an audience perspective. For example, in Sonn et al.’s (2015) research, the authors were left pondering the audience impacts of the counter‐narratives presented by African Australian youth to their local police, the latter of whom were complicit in endorsing a narrative that criminalized the youths’ existence.

Extending on the ‘window’ metaphor introduced earlier (Hughes‐Hassell, 2013), one must be careful not to simply be a spectator to another’s story and to instead move beyond the voyeurism that commodifies difference. In AMKA, white audiences are those who are in this “window” position; they are the ones who support and benefit from the public discourses that legitimize their existence and vilify African Australians. As such, the current research is important in understanding how white spectators are interpreting the performance and what implications these interpretations have for dismantling whiteness.

## Methodology

### Research Design

The epistemological position underpinning the current study is one of social constructionism. This epistemology emphasizes both the contextual embeddedness of knowledge as well as the constantly shifting nature of this context not only across time but across social interactions (Burr, 1995). As meaning‐making in art is unique to each individual who interacts with the art product, the investigation into what impacts are experienced by audiences is quintessentially a social constructionist endeavor.

In the current project, it was acknowledged that audiences interpreted and responded to AMKA through their own lenses which consist of both personal experience (micro) and the historical, societal, political, and cultural settings (macro) in which the performance was witnessed. However, these micro and macro sense‐making processes are not insular – there is a bilateral influence whereby “society and persons are interwoven” (Sonn, Stevens, & Duncan, 2013, p. 303) to alter the way in which the self is constructed through interpersonal interaction and the way in which the dominant narrative is presented at the structural level. Through this interaction, one can work at the level of everyday narrative to understand, critique, and transform the institutional discourse, as is posited by a critical narrative approach (Souto‐Manning, 2014) and critical studies of race and whiteness (Green et al., 2007). It is in using this approach that the current research project aimed to elucidate how contemporary narratives around Africans in Australia are constructed by focusing on the articulation of these narratives at the level of the individual audience member.

Additionally, the current research is guided by a theoretical framework of critical whiteness scholarship (Green et al., 2007). Whiteness is directly implicated in the maintenance of marginalizing narratives; it silences and discredits alternative ways of knowing (Smith, 1999) by controlling public discourse to legitimize and reproduce white privilege. However, discourse itself can be transformed for liberation‐oriented purposes as it both shapes, and is shaped by, the culture in which it functions (Freire, 1972; Reenskaug Fjørtoft, 2013). Understanding the processes and possibilities of discourse involves examining how it is used and reproduced at the individual level of story and text. Guided by these epistemological and theoretical positions, a post‐performance open‐ended survey design often used to assess audience impact was selected for data collection (Appendix S1).

### Participants

The current study is a subset of a larger research project conducted in partnership between cohealth and Victoria University (Sonn, Agung‐Igusti, & Komba, 2018). As such, the data used in this study are drawn from a larger survey of AMKA attendees. There were four AMKA performances scheduled in September 2017, all held at a prominent performing arts center in Melbourne. One performance was exclusive to high‐school students while the other three were open public performances. All adult (18 years of age and over) audience members who attended one of the public performances were approached for participation. Due to methodological constraints, demographic data were only collected for those who self‐selected be a part of the study, rather than for all AMKA attendees. In line with the research aims, only those identifying as white were included in the current study. White respondents were considered those who either identified themselves as such or who spoke about their whiteness in their responses.

One hundred and sixteen responses were collected, with 21 responses being removed from the final count due to participants electing not to provide consent or not providing enough information to yield meaningful data. Of the remaining 95 audience members who completed the survey, 34 were categorized as having a white cultural background. Two thirds of these spectators identified as female (*n* = 23) with the remaining third identifying as male (*n* = 11). None of this sample fell within the age range of 18 to 24 years old (as compared to 24% of the overall sample), 29% were between 25 and 34 years old, 29% were between 45 and 54 years old, 24% were 35 to 44 years old, 6% were 55 to 64 years old, 3% were 65 to 74 years old, and 9% were 75 or older. Twenty‐six participants were employed, four were retired, two were unemployed, one was a student, and one reported their occupation status as “other.” Most of these spectators had attended the arts venue previously (94%) and felt comfortable in the space (74%).

### Procedure

Ethics approval for the current study was obtained from both the Victoria University Human Research Ethics Committee and the cohealth Human Ethics Advisory Group. Data were collected using mixed open‐ and closed‐ended surveys. Demographic information was first collected, consisting of participants’ age, current occupation, gender, cultural background, and residing suburb. The remainder of the questionnaire utilized a free‐response format to investigate participants' reactions to, and interpretations of, AMKA. These questions asked participants about their understandings of arts outcomes in general, motivations for attending AMKA, the most memorable or impacting aspect of the performance and why this aspect had such an effect, any learnings experienced as a result of spectating, and how they would share their experience of AMKA with others.

At the conclusion of each performance, spectators were informed by a cohealth liaison about the research project and the process for opting‐in for participation, as well as provided with an information sheet about the project. Paper copies of the surveys were distributed alongside website links for those who wished to complete the survey electronically (accessed via Qualtrics). The time taken to complete the questionnaire was not expected to exceed 20 minutes. For a month following the performances, the cohealth Facebook page posted reminders to encourage audience members to complete the surveys. Participants varied in their response detail, from opting to leave questions unanswered or providing only a few words, to writing multi‐sentence paragraphs. The level of detail in the responses did not appear to differ across the survey form (i.e., online or paper), although most (*n* = 29) of the responses were collected via the internet survey.

### Data Analysis

The data obtained from the surveys were analyzed using NVivo (version 11). Thematic analysis was then employed, a flexible method of exploring data that involves developing themes through the analysis of patterns (Braun & Clarke, 2006). To begin, the data were imported into the program and then read several times to facilitate familiarization of the content. The initial reading was guided by the research questions and was thus more inductive (Braun & Clarke, 2006). This stage involved making annotations against the survey responses and constructing patterns of recurring themes. Initial codes were created and then categorized into encompassing themes and sub‐themes (i.e., “nodes” in NVivo) which were refined through an iterative process of reading and re‐reading the text. Themes were examined, modified, and removed where necessary to facilitate clarity and persuasiveness of their ideas. Handwritten notes and thematic maps accompanied NVivo analyses to assist in the creation and structuring of themes. Data analysis was concluded when data saturation had been reached and re‐readings of the data revealed no new insights.

## Results

Two themes were developed in response to the research questions about the meanings and experience of implicated witnessing for ostensibly white spectators: *understanding the counter‐narrative in context* and *engaging in reflective practices*. Each theme contained several sub‐themes which aimed to provide further elucidation on what it meant to be an audience member in this space.

### Understanding the Counter‐Narrative in Context

This theme refers to how audience members were cognisant of the larger structures in which the counter‐narrative was enacted. In this way, spectators interpreted the piece not as an isolated performance but as commentary connected to events outside the theater walls, as art reflecting everyday life. Their responses were categorized into three contextual levels: *the aesthetics and the counter‐narrative, outsiders on the inside,* and *reflections on political realities*.

#### “Art is a Powerful Tool”: Aesthetics and the Counter‐Narrative

I felt that slot [sic] of time and effort was put into the communication of the message. It was heartfelt and well executed. (Male, 35‐44 yo.)

The power in the performative counter‐narrative comes not just in the construction of the narrative itself, but also by its *delivery*. For AMKA, the art forms, the venue, the production quality, the performers, and the special effects all worked together to enhance the receptivity of the narrative. One audience member reported that the characteristics of the venue and the simplicity of the performance facilitated her feeling of immersion in, and connection to, the narrative:

The production, which was most interesting in the fact that it was so focused, and with so little embellishment yet it was able to keep one intently interested in the performance… The fact that I became so involved in the stories told, that the passage of time was fleeting. The accoustics [sic] of the venue, [arts centre], were such that one was able to be involved in the performance, and offered no distractions at all. (Female, 75 + yo.)

By selecting a professional and comfortable location in which to present the counter‐narrative, spectators were able to concentrate on sense‐making and interpretation as opposed to having to contend with extraneous, attention‐diverting stimuli that distract from sustained engagement. Similarly, by making restrained decisions about the production, audiences were not overwhelmed with having to process an excessive amount of information and instead were masterfully directed through the piece according to a clear aim.

Also reported by spectators as contributors to this feeling of immersion included the rapport between, and professionalism of, the performers, the staging and set design, the use of props, and the diversity of art mediums utilized. For instance, responses to the questions “What stood out about this performance?” and “Why did this stand out?” included: “personal narratives combined with excellent lighting and design”, “the movement was an effective tool, and I enjoyed the visual imagery, non‐verbal communication and the pace the movement gave to the performance”, and “… the talent of the performers and the quality of sound production really stood out‐ this really drew me into the performance”. Together, these characteristics encouraged strong emotional responses through embodiment of the narrative. In fact, “powerful” was the most cited descriptor of the performance (15 instances), with one spectator noting “that art is a powerful tool for deconstructing assumed knowledge about 'race' and social difference.” It can be speculated that this intense, impactful feeling was facilitated by the unobtrusive production elements that simultaneously drew people into the performance while creating a space in which engagement with the content could be optimized. Alternatively, however, a singular focus on the technical aspects of the performance may be indicative of a distancing strategy used to disengage from processing the counter‐narrative at a more critical level. Instead, these spectators may be using the performance as an opportunity for entertainment only or voyeurism into lived experiences that differ from their own.

#### “A Show that Normally Doesn’t Get Shown”: Outsiders on the Inside

Although the venue was revered for its conveniences from an aesthetic perspective, spectators were also aware of its role in racially encoding space. Audience members described AMKA as being a “unique” and “unusual” event in this setting as African Australian communities were categorized as separate to the usual clientele catered for by this arts center (that is, white). Further, spectators reflected on the importance of disrupting the absence of these narratives in the public discourse, commenting that AMKA presented “new stories on the theatrical stage”, gave audiences the opportunity to “see a show that normally doesn't get shown”, and amplified the “voices of people who don’t get airtime in this arena.”

Audience members were also cognisant of how other people in the venue interacted with each other and the space. For instance, one spectator noted that the space was patrolled by white staff who behaved in ways that conflicted with the counter‐narrative being presented and instead reiterated the whiteness, privilege, and power inherent in the venue:

I also learnt that I get very cranky with over zealous arts center staff who would not allow late comers to enter unless at specific times and was quite difficult to deal with. The irony of a white man telling community what to do was not lost that day. It wasn't empowering to watch. (Female, 35‐44 yo.)

This observation is a reminder that although the African Australian community had gained access to a white space as both performer and audience, this was only a temporary disruption and one which was monitored by whiteness. The respondent interpreted this behavior as disempowering, emphasizing that the space remained subject to control by the white staff who, as representatives of an institution, granted them access. Spaces are not neutral; they are racially encoded and embedded in power (Barajas & Ronnkvist, 2007; Leitner, 2012; Sullivan, 2006) and they are more than simply a convenient location in which to communicate a narrative. These spaces systematically change the way in which the audience makes sense of the narrative. Consistent with Teo’s (2018) argument that art can assist in “reclaiming public spaces as an act of resistance” (p. 261), AMKA was perceived by some spectators as beginning to diversify white spaces while exposing the racist structures that uphold them.

#### “The Unsettling Reality of Current Times”: Reflections on Political Realities

Consistent with the aims of counter‐storytelling, some audience members realized that the narratives presented in AMKA contrasted with those that were publicized by the media and by politicians, with one spectator noting that the most impactful part of the performance was:

The stories both individually and collectively of the performers… Because they are hidden, silenced and distorted in the colonised structures of white mainstream society. (Female, 25‐34 yo.)

This audience member acknowledged the deliberate concealment of African Australian narratives and attributes these actions to the oppressive history underlying contemporary Australian society which continues to be felt. In rejecting these partial discourses, spectators appeared to turn to the alternative narratives offered by AMKA as more truthful portrayals of reality. The narrative that they constructed in response to the one that was performed is more holistic than the current cultural narrative, including both an acknowledgment of struggle and challenge while also leaving space for imagining counter‐possibilities of strength, hope, and resilience:

The words and the delivery were extremely powerful. They so powerfully communicated the harm done to black bodies/minds, communities, yet ultimately it was a reclaiming of history, identity, culture… (Female, 25‐34 yo.)

Spectators also observed parallels between the counter‐narrative and other marginalized groups in Australia, remarking how the performance revealed “the unsettling reality of current times” and that “this is the story of one community, but no doubt there are variations on the same theme among many different communities who cal [sic] Australia home.” As a result, these audience members were engaging in multiple levels of analysis – they were aware of each individual performer’s story, the shared performance narrative of Africans in Australia, and a shared communal narrative encompassing both African Australians as well as other communities who have been marginalized and vilified due to their cultural background.

One spectator mused upon the similarities between the experiences of the African Australian community and Aboriginal and Torres Strait Islander people, another made connections to refugee populations seeking safety in Australia, while others acknowledged that anyone coded as “newly arrived” or as a “person of color” could be subject to white Australia’s intolerance and unresponsiveness. As such, everyday and systemic injustice was perceived as a bond for communities who have been relegated to the margins of society.

At the same time, however, some spectators were able to acknowledge the complexity and the diversity of lived experience between (and within) African Australian communities and those groups who have been othered. In this way, audience members were able to use AMKA as a springboard for considering issues of whiteness, belonging, coloniality, and racism more broadly. The following quotation reflects a spectator grappling with this idea of belonging at multiple levels – for themselves personally and at a national level:

Belonging is such an incredible theme for this land/country/Australia. I mean who really belongs here anyway? My ancestors arrived in 1850s but I don't really think that means I have more entitlement than people newer to this land ‐ it just means I am more comfortable here really. (Female, 45‐54 yo.)

This spectator connects the personal to the political by deliberating on how belonging is tied into identity politics. Through a consideration of Australia’s historical and continued colonization, this spectator takes the conversation initiated by AMKA to a national level, contemplating the implications for peoples’ right to land and belonging.

### Engaging in Reflective Practices

This theme contains references to altered knowledge about the self via turning one’s gaze inwards and critically appraising one’s own positionality and responsibility regarding the existing narrative around African Australians. Responses are organized into three sub‐themes: *the discomforting process of confronting privilege, seeing what one has been taught not to see,* and *theater as a reminder and prompt for action*.

#### “It Did Make Me Feel Uncomfortable in a Couple of Spots”: The Discomforting Process of Confronting Privilege

Some audience members found that the performance facilitated insight into their own behaviors, beliefs, attitudes, and privileges. Connections were made between the content and personal experiences, relationships, and/or workplaces, with one spectator responding that they learned:

The structures of my workplace are racist yet clouded by the role of 'doing good' … and 'working with' people of colour, the absence of these conversations that take place, naming it for what it is‐ white privilege, white guilt, white saviours, do‐gooders. (Female, 25‐34 yo.)

While another drew parallels between a scene in the performance and their own day‐to‐day experiences:

The bit about how other people on the train perceive young black men joking and laughing and "fooling around" on the train made me think about how I may perceive and show judgement in situations like that. (Male, 35‐44 yo.)

This response illustrates a growing awareness of how one’s behavior may be guided by a dominant culture narrative that portrays African Australian men as disruptive and disorderly. They began to question the norms to which they had become accustomed, the norms which operate to their advantage by validating their identity and their entitlement to space and belonging. In this way, several spectators appeared to shift in their knowledge about themselves as they began to understand how their own behavior, and the behavior of those around them, upholds and conforms to oppressing narratives.

Accompanying the engagement in these processes were feelings of being unsettled, with one spectator remarking that “it did make me feel uncomfortable in a couple of spots and not all theater does that”. This discomfort appeared to result from turning the gaze inwards to critically examine one’s own privilege, with many spectators making reference to their privilege when answering what they learned about themselves from witnessing AMKA: “That checking my privilege is important”, “Continuing to understand my privilege in different settings and reflecting this within my professional work as well as my personal relationships with my family and friends”, “I felt empathy at the same time as realising the great privilege I have”, and “I am uncomfortable about my privilege [sic] and I do not know what it is like to walk in the shoes of others”.

Although confronting privilege can result in defensive or paralytic reactions that are counterproductive to personal and societal change, some spectators instead declared intentions for post‐performance actions provoked by a new sense of responsibility. One audience member outlined her intentions as follows:

How much more I have to learn, give space for voices that aren't mine, get out of the way of great talent, understand the depth of pain. (Female, 45‐54 yo.)

In this response, the audience member appears to record intentions for depowerment (Huygens, 1997) by voluntarily removing herself from her position of privilege to equalize the stark discrepancies in power. By giving up rather than taking up space, this spectator seems to acknowledge her power in being the one who can allocate space. Although these responses are only intentions and not indicative of tangible change, audience members who assumed actionable positions in relation to the narrative demonstrated evidence of becoming implicated witnesses.

Several spectators, however, appeared not to engage in self‐awareness exercises. These audience members avoided using first‐person language and positioning themselves as subjects when providing responses. This deliberate distancing from implication may reflect a disconnecting strategy, with engagement of the narrative only occurring at superficial levels. Only one spectator explicitly disengaged with the narrative, displaying the emotional retaliation typically associated with being confronted with one’s privilege:

… I do not get anything from a piece of theatre when the actors are only playing aggression to get their point across. (Male, 45‐54 yo.)

This spectator appears to invalidate the lived experiences of the artists, denying them the right to be angry about their struggles of living in a white‐dominated culture. In addition, AMKA was a complex performance that spoke not only of anger and struggle, but also of hope and strength. By selectively focusing on the anger, this spectator might have been filtering the performance through the lens of the dominant cultural narrative (which depicts African Australian communities as violent and aggressive) and seeking out confirmatory evidence for the truthfulness of the narrative.

#### “I Know Nothing”: Seeing What One Has Been Taught Not to See

In this sub‐theme, white audiences began to think in new ways, broaden their perspectives, and exhibit humility in acknowledging the inadequacies of their current understandings. Some spectators came to question their partial knowledges and how they have been sheltered by ignorance until confronted with the reality of lived experience through AMKA. As illustrated by the quote below, with no incentive to otherwise engage in these topics, white audiences indulge in the comfort of not knowing:

My white privilege in learning about these stories and experiences when people of colour have no choice, they live these experiences daily. (Female, 25‐34 yo.)

As what has been hidden from them is revealed, white audiences started to become aware of the incompleteness of their current lenses which was succinctly articulated by one participant as “I know nothing”. Some of the spectators were already aware of their limited perspectives prior to the performance, noting in several cases (*n* = 9) that their motivations for attending AMKA were to “learn about the African Australian experience”. The performance was therefore seen as an opportunity to unlearn, with the artists seemingly being positioned as the educators in charge of correcting these biases.

However, some audience members realized that the performance was not created for their needs. Despite being an opportunity for learning, it was also acknowledged that “actors were there to speak for themselves” and “it [AMKA] didn’t try to be anything other than what it wanted to be”. In these statements, some spectators showed understanding that the performance was created by and for the artists and not to placate the white audience. Similarly, although audience members saw the theater as an opportunity to be “taken inside the skin of a community I’m not part of”, they also reported limitations to perspective‐taking processes as a result of their positions of privilege:

If I'm inclined to forget the fact, that there is much that I don't know and can't even imagine. (Female, 55‐64 yo.)

The consequence of this revelation is that perhaps rather than reducing the felt social distance between the white audiences and the performers, the chasm of difference increased. That is, as white audiences became aware of the struggles faced by African Australian communities, they may have felt more distant to the “other”:

…there is a bigger distance between me and the lived experience of the performers and perhaps by association African community more broadly than I might have assumed. (Male, 45‐54 yo.)

#### “It’s Important to Hear Information Again”: Theater as a Reminder and Prompt for Action

Several spectators reported that rather than learning anything new about themselves or dissimilar others, the performance served to encourage the continuation of current behaviors, beliefs, and/or attitudes. For instance, one audience member responded that for her:

It wasn't so much a learning, but a reminder of the way in which racism/whiteness harms people and communities. (Female, 25‐34 yo.)

Comments like this one suggest that spectators believe they are already aware of how whiteness is implicated in disadvantage. AMKA, therefore, is perceived as a confirmation of established knowledges and an additional source of evidence showing the extent and continuation of the injustices. However, audience members for whom this theme applied also responded that this reiteration reinvigorated action:

It reminds me how important it is to continue to better inform myself and fight for a safe workplace environment to peel these layers back and continue to align my practice with a structural perspective. (Female, 25‐34 yo.)

In this way, these spectators demonstrate a responsibility for their witnessing not only for themselves, but for their communities and workplaces as well. Similarly, another spectator commented that “it's important to hear information again; to understand the scale on which it's felt throughout the community”, indicating that the performance was used to provide confirming evidence that incidents of race‐based violence are not isolated but part of a larger structural issue. Far from being a redundant consequence of spectating, Sajnani (2010) states that “people continue to need reminders that they can effect change” (p. 193). Consequently, AMKA reminded the audience of the continued need to engage in critical reflexivity while also reiterating the capability and responsibility of one to act.

Alternatively, these comments may reflect a stagnation in reflexivity, such that audience members may feel that they have reached some arbitrary point of awareness in their white privilege that they deem sufficient, making no further involvement necessary. Critical race scholars critique this idea that such an endpoint exists, and that by entertaining the belief that one has achieved some arbitrary pinnacle is in fact counterproductive to efforts at dismantling whiteness (Mezirow & Taylor, 2009). For example, the following quotation appears to indicate that the spectator’s current actions were considered adequate:

I learnt that the way I am and my tolerance is important in the growth of humanity. (Male, 35‐44 yo.)

Bypassing discussions about the inadequacy of tolerance as a benchmark for social justice (refer to Green et al., 2007 for a critique), the spectator’s language here indicates a closing off from conversations around continued learning and personal growth. While acknowledging his responsibility in social change, this spectator avoids implicating themselves and their whiteness. This wilful disconnection exacerbates inequalities in risk‐taking and vulnerability as audiences remain passive consumers rather than active witnesses. Spectators voyeur into the life of the “other,” seeking understanding outwards but avoiding understanding of the self. If there is no critical reflection accompanying viewing, then the performative counter‐narrative risks commercializing and exoticizing cultural differences for the viewing pleasure of the white audience (Sonn & Quayle, 2013; Sullivan, 2006).

## Discussion

The current research investigated the meaning‐making processes for white audiences in a performative counter‐storytelling piece by Africans in Australia called AMKA. Using post‐performance surveys and thematic analysis, audience member responses showed that receptivity of performative counter‐narratives and the experience of implicated witnessing can be understood through the complex interaction of many factors: the individual factors particular to each audience member, the context in which the counter‐narrative is performed, the interaction between the audience and the performers, and the quality of the production.

Artist intentionality is an important aspect of art creation, however, once opened to the public, the art is involved in a continued state of becoming and negotiation of meaning with the audience (Conner, 2004). This meaning‐making can occur at varying levels of depth, with some AMKA spectators surpassing superficial interpretations of the performance to instead seek out more meaningful connections with the art and the narrative as implicated witnesses (Sajnani, 2012). For part of this white audience sample, the content appeared to present new perspectives, while for others it functioned as a timely reminder not to become complacent. In this way, extending upon Hughes‐Hassell’s (2013) window/mirror metaphor, a mirror could also be said to be held up to the white audiences so that they could observe what has been “hidden” from them, but that which is plain to see by those who are harmed by privilege. Longitudinal research methods are required to comprehend the extent of these impacts, particularly regarding their effects across time as a part of a continuous journey of unlearning and critical reflexivity.

Shifts in the relationship with the “other” were also observed; the “space of encounter” (Mayblin et al., 2016, p. 216) created during AMKA appeared to result in an awareness about and amplification of the social distance between lived experiences as spectators acknowledged the gulf of not knowing that their privilege afforded them. It is possible that community art projects that report bridges being developed across socially diverse groups over the course of art production first grapple with this awareness of different lived experiences. These projects (e.g., Madyaningrum & Sonn, 2011; Sonn et al., 2014) discuss reduction in social distance as the result of “contact zone” creation (Pratt, 1991, p. 34) whereas the inhabitation of the implicated witness identity is temporary within the theater space. These results should not undermine the importance of the theater as an opportunity for facilitating intercultural exchange, rather it reiterates that attending a singular performance is an act of “showing up ”, supporting, and responsible listening within a lifetime of allyship intentions.

This relationship was also altered for some participants into an educator/student dynamic. On the theatrical stage and in society more broadly, minority groups are often tasked with educating privileged populations about their lived experiences (a form of “cultural taxation”; Padilla, 1994, p. 26). Similarly, whiteness is re‐enacted in the privilege of white audience members requiring constant reminders in order to remain attentive to inequalities from which they benefit. This positioning of “other as educator” has implications for who has the responsibility to teach and to incentivize white people to take an interest in anti‐racist efforts. Further, although this positioning could be interpreted as a sign of respect, of acknowledging one’s limited perspectives and valuing another’s knowledges, it can also position white audiences as the subjects and the knowers, while simultaneously positioning the “other” as the object and the known (Green et al., 2007). That is, African Australian communities may be positioned as those about whom one can obtain knowledge, while the white audiences are the active and autonomous agents. This process of positioning reproduces inequalities in the relationship between vulnerable and privileged groups by further disempowering the former.

## Limitations

The participants in the current research may have been biased toward critiquing their privilege and deconstructing dominant narratives as a result of their self‐selecting status to both the performance and the research project. Spectators with negative reactions to the performance may have opted out of participating or individuals with strong beliefs in the current dominant narrative around African Australians may have avoided the performance altogether. For instance, in responding to the question, “What would you tell others about this performance?”, one audience member answered with, “The people I know would not be interested!!”. Additionally, over half of the responders in the white‐identifying sample (*n* = 19) commented that they had decided to attend the performance because they knew the performers directly or had connections with cohealth Arts Generator. These observations have implications for questions around who is listening, who needs to listen, and how we reach them. Answering these questions was beyond the scope of the current research but is an important avenue for future investigation, as voiced by others (Sajnani, 2012).

## Conclusion and Future Directions

This research has contributed to the wealth of existing literature recommending community arts as a valuable vehicle for social change. It has extended upon this literature by addressing the gap of audience responses to performative counter‐narratives and by turning the focus from art producers to art spectators, implicating the latter in the pursuit of social justice. A focus on advantaged groups is necessary as the dismantling of racism and other forms of systemic injustice requires those in privilege to actively be involved in critical thinking and depowering processes. Assisted by artistic processes which help to facilitate involvement with the narrative by their embodied and provocative qualities, the performative counter‐narrative does not provide solutions but instead acts as a catalyst for further action and reflection. This reflection is a part of a lifelong journey of anti‐racist allyship commitment, defined by questioning, vigilance, and working toward personal and structural transformation.

A surprising finding in the current research was the significance of production quality in determining receptivity of the counter‐narrative. Perhaps it is not enough for a counter‐narrative to be communicated creatively to be effective, but that the communication method must also be of a sufficiently high standard. Although art research discusses the importance of ensuring quality in the presentation of an art piece (e.g., Radbourne, Glow, & Johanson, 2010), there has not yet been a parallel discussion within the performative counter‐storytelling literature. This finding should be subject to further investigation as an enhanced understanding of how the production of a counter‐narrative affects audience responses could assist in expanding the reach of a performance to those who wilfully disengage from a position of responsible listening.

Community psychology researchers can encourage positions of responsible listening through the adoption of research methods that ask targeted, reflective questions post‐performance or create spaces for intercommunal dialogue (Watkins & Shulman, 2008). Research tools can continue the theater experience beyond its formal conclusion by allowing participants to process their experience at deeper levels beyond a consideration of mere satisfaction levels (Radbourne et al., 2010).

Although it cannot be claimed that a single performance has the power to revolutionize deeply embedded structural inequalities (Madyaningrum & Sonn, 2011; Sonn et al., 2015), the impacts that are observed at the level of the individual audience member are by no means inconsequential. Through the integration of aesthetic excellence with expressions of lived experience, the community arts are a powerful tool for enabling counter‐possibilities of hope, resilience, and justice to both be imagined and realized.

## Conflict of Interest

All authors declare no conflict of interest related to this study.

## Supporting information


**Appendix S1** Audience Questionnaire.

## Footnotes

^1^
The artists involved in AMKA self‐identify as Africans in Australia as opposed to African Australian as commentary on belonging and identity within Australia.
