# Supplementary material for: The Performative is Political: Using Counter‐Storytelling through Theater to Create Spaces for Implicated Witnessing
Source: Am J Community Psychol. 2020 Dec 22;68(1-2):47–60. doi: 10.1002/ajcp.12493 (PMC8672364; doi:10.1002/ajcp.12493)
Supplement: Supplementary file 1 — Appendix S1 Audience Questionnaire. [file AJCP-68-47-s001.docx]

**Supplementary Materials: Audience Questionnaire**

1. What is your preferred gender? [open-response]
2. What is your age? [closed-response: 18-24, 25-29, 30-34, 35-39, 40-44, 45-54, 55-69, 70-74, 75+]
3. What is your cultural background? [open-response]
4. What is your current occupation? [closed-response: student, employed, unemployed, retired, other]
5. What is the suburb you live in? [open-response]
6. What is your understanding of the purpose of arts? [open-response]
7. Have you been to the arts centre before? [closed-response: Yes/No]
8. How comfortable do you feel being at the Arts Centre? [closed-response: very comfortable, somewhat comfortable, neither comfortable or uncomfortable, somewhat uncomfortable, very uncomfortable]
9. Why did you come to see the performance? [open-response]
10. What stood out for you the most? [open-response]
11. Why did this stand out? [open-response]
12. Through witnessing this performance, what did you learn:
    1. About yourself? [open-response]
    2. About your community and different people within it? [open-response]
13. Would you come again to this type of performance? [closed-response: Yes/No]
14. Would you come again to the Arts Centre? [closed-response: Yes/No]
15. What would you tell others about this performance? [open-response]
